# Specific inhibition of the NLRP3 inflammasome suppresses immune overactivation and alleviates COVID-19 like pathology in mice

**DOI:** 10.1016/j.ebiom.2021.103803

**Published:** 2021-12-31

**Authors:** Jianxiong Zeng, Xiaochun Xie, Xiao-Li Feng, Ling Xu, Jian-Bao Han, Dandan Yu, Qing-Cui Zou, Qianjin Liu, Xiaohong Li, Guanqin Ma, Ming-Hua Li, Yong-Gang Yao

**Affiliations:** a Key Laboratory of Animal Models and Human Disease Mechanisms of the Chinese Academy of Sciences & Yunnan Province, KIZ-CUHK Joint Laboratory of Bioresources and Molecular Research in Common Diseases, Kunming Institute of Zoology, Chinese Academy of Sciences, Kunming, Yunnan 650201, China; b Kunming National High-level Biosafety Research Center for Non-Human Primates, Center for Biosafety Mega-Science, Kunming Institute of Zoology, Chinese Academy of Sciences, Kunming, Yunnan 650107, China; c National Resource Center for Non-Human Primates, National Research Facility for Phenotypic & Genetic Analysis of Model Animals (Primate Facility), Kunming Institute of Zoology, Chinese Academy of Sciences, Kunming, Yunnan 650107, China; d Kunming College of Life Science, University of Chinese Academy of Sciences, Kunming, Yunnan 650204, China; e CAS Center for Excellence in Brain Science and Intelligence Technology, Chinese Academy of Sciences, Shanghai 200031, China

**Keywords:** SARS-CoV-2, NLRP3 inflammasome, Immune overactivation, Cytokine storm, MCC950

## Abstract

**Background:**

The Coronavirus Disease 2019 (COVID-19) pandemic has been a great threat to global public health since 2020. Although the advance on vaccine development has been largely achieved, a strategy to alleviate immune overactivation in severe COVID-19 patients is still needed. The NLRP3 inflammasome is activated upon SARS-CoV-2 infection and associated with COVID-19 severity. However, the processes by which the NLRP3 inflammasome is involved in COVID-19 disease remain unclear.

**Methods:**

We infected THP-1 derived macrophages, NLRP3 knockout mice, and human ACE2 transgenic mice with live SARS-CoV-2 in Biosafety Level 3 (BSL-3) laboratory. We performed quantitative real-time PCR for targeted viral or host genes from SARS-CoV-2 infected mouse tissues, conducted histological or immunofluorescence analysis in SARS-CoV-2 infected mouse tissues. We also injected intranasally AAV-hACE2 or intraperitoneally NLRP3 inflammasome inhibitor MCC950 before SARS-CoV-2 infection in mice as indicated.

**Findings:**

We have provided multiple lines of evidence that the NLRP3 inflammasome plays an important role in the host immune response to SARS-CoV-2 invasion of the lungs. Inhibition of the NLRP3 inflammasome attenuated the release of COVID-19 related pro-inflammatory cytokines in cell cultures and mice. The severe pathology induced by SARS-CoV-2 in lung tissues was reduced in *Nlrp3*^−/−^ mice compared to wild-type C57BL/6 mice. Finally, specific inhibition of the NLRP3 inflammasome by MCC950 alleviated excessive lung inflammation and thus COVID-19 like pathology in human ACE2 transgenic mice.

**Interpretation:**

Inflammatory activation induced by SARS-CoV-2 is an important stimulator of COVID-19 related immunopathology. Targeting the NLRP3 inflammasome is a promising immune intervention against severe COVID-19 disease.

**Funding:**

This work was supported by grants from the Bureau of Frontier Sciences and Education, CAS (grant no. QYZDJ-SSW-SMC005 to Y.G.Y.), the key project of the CAS “Light of West China” Program (to D.Y.) and Yunnan Province (202001AS070023 to D.Y.).

**Research in Context**

**Evidence before this study**

COVID-19 patients mainly experience two disease processes including lung pathology and dysregulated immune response, the latter further aggravating lung damage and potentially affecting other organs. Specifically, the lack of knowledge on how SARS-CoV-2 interacts with host immune system prevents the development of antiviral drug especially regarding the suppression of immune overaction in severe COVID-19 disease. It has been reported that cytokine storm plays a key role in COVID-19. A most recent study showed that SARS-CoV-2 N protein promoted NLRP3 inflammasome assembly to contribute to the maturation of pro-inflammatory cytokines most from *in vitro* experiments.

**Added value of this study**

We provided multiple lines of evidence that NLRP3 inflammasome plays an important role in host immune response to SARS-CoV-2 invasion. SARS-CoV-2 infection induced NLRP3 inflammasome activation to release IL-1β and IL-18 *in vitro* and *in vivo*, priming a systematic immune response, which in turn aggravates lung immunopathology in murine models. Suppression of NLRP3 inflammasome by genetic knockout and specific inhibitor in the mouse models consistently displays its immune importance in COVID-19 disease progression. Specific NLRP3 inflammasome inhibitor MCC950 effectively alleviates SARS-CoV-2 induced immune activation and immunopathology in a mouse model.

**Implications of all available evidence**

The evidence from *in vivo* studies highlighted that inflammatory overactivation induced by SARS-CoV-2 is a main stimulator of COVID-19 related lung immunopathology. We provided previously unknown insights into how SARS-CoV-2 interacts with host immune response within COVID-19 disease, supporting the potential of targeting NLRP3 inflammasome as immune intervention against COVID-19 disease.

Alt-text: Unlabelled box

## Introduction

The Coronavirus Disease 2019 (COVID-19) pandemic has been a global crisis and caused much more devastation than other infectious diseases.^1^,^2^ The pandemic is caused by a virus, a member of the *Coronaviridae* family, now known as severe acute respiratory syndrome coronavirus 2 (SARS-CoV-2).^3^, ^4^, ^5^, ^6^, ^7^, ^8^ The high mortality from SARS-CoV-2 is associated with severe respiratory dysfunction. Coronaviruses use a spike glycoprotein (S) to find a receptor before cell membrane fusion and virus entry. Like SARS-CoV, SARS-CoV-2 uses angiotensin-converting enzyme 2 (ACE2) as a receptor for cell entry.^6^^,^^9^, ^10^, ^11^ For most COVID-19 patients, the symptoms are mild, often only with a low fever and cough, although about 15% of them also have some breathing difficulty.^2^,^12^,^13^ However, severe COVID-19 infection is the result of developing an acute respiratory distress syndrome (ARDS), with severe pulmonary injury probably caused by the dysregulated excessive inflammation in the lungs.^14^, ^15^, ^16^, ^17^, ^18^ Nevertheless, how SARS-CoV-2 interacts with host immune system and finally causes lung immunopathology remains largely unknown.

An array of pathologies including lymphopenia, neutrophilia, dysregulation of monocytes and macrophages, reduction or delay of type I interferon response, and cytokine storm have been diagnosed in COVID-19 patients.^2^,^16^,^17^^,^^19^, ^20^, ^21^ There is an increasing body of evidence suggesting that these immunopathological processes play a critical role in COVID-19 pathogenesis.^22^,^23^ Specifically, such immunopathology is characterized by immune overactivation with the hyperproduction of an array of pro-inflammatory cytokines; and the extent of the overproduction has been shown to correlate adversely with the prognosis.^24^ The excessive secretion of pro-inflammatory cytokines initiates multiple inflammatory signaling pathways via their receptors on target cells, causing a series of complicated symptoms and dysfunction of innate or adaptive immunity against the SARS-CoV-2 pathogen.^25^ The elevated levels of multiple cytokines including IL-1β, IL-2, IL-6, IL-7, IL-8, IL-10, IL-12, IL-17, IL-18, TNF-α, MCP-1, G-CSF, GM-CSF, IP-10, and IFN-γ have been reported in COVID-19 cases with severe symptoms.^26^ Thus, the correlation of COVID-19 severity with the excessive pro-inflammatory status calls for an urgent study into developing immune interventions against COVID-19 disease in parallel with global vaccination programs.

The NOD-like receptor protein 3 (NLRP3) inflammasome comprises the NLRP3 sensor, caspase-1, and the adaptor molecule apoptosis associated speck-like protein containing a caspase recruitment domain (ASC).^27^ When activated, matured caspase-1 cleaves pro-IL-18 and pro-IL-1β into mature IL-18 and IL-1β, respectively, which are then secreted.^28^,^29^ Upon sensing a wide range of microbial motifs, endogenous danger signals and environmental irritants, NLRP3 inflammasome causes the release of proinflammatory molecules including IL-18 and IL-1β to further expand host immune response, which is usually correlated with multiple disease severity.^27^,^30^,^31^ NLRP3 involvement in COVID-19 has been demonstrated by the following lines of evidence: (1) the NLRP3 inflammasome responds to SARS-CoV-2 infection.^32^,^33^ and is associated with COVID-19 severity in patients;^34^, ^35^, ^36^, ^37^ (2) NLRP3 inflammasome activation is associated with the COVID-19 related cytokine storm^37^,^38^ and is activated by the SARS-CoV-2 N protein;^39^ (3) age-induced NLRP3 inflammasome over-stimulation increases lethality of SARS-CoV-2 pneumonia in elderly patients;^40^ and (4) SARS-CoV-2 infection in blood monocytes leads to activation of the NLRP3 inflammasome and contributes to the formation of COVID-19 related cytokine storm.^41^ However, whether the NLRP3 inflammasome is directly involved in the severe COVID-19 pneumonia or a byproduct of the SARS-CoV-2 infection remains to be resolved.

In the present study, we have characterized the role of NLRP3 in SARS-CoV-2 infection by using *in vitro* and *in vivo* models. We have shown that inhibition of the NLRP3 inflammasome reduced the release of pro-inflammatory cytokines in SARS-CoV-2 infected immune cells. *Nlrp3* deficiency suppressed the pro-inflammatory response and thus reduced SARS-CoV-2 induced lung pathology in a mouse model. The use of small molecule MCC950, a specific inhibitor of NLRP3 inflammasome activity,^42^ alleviated COVID-19 like pathology in human ACE2 (hACE2) transgenic mice. These results demonstrated that targeting the NLRP3 inflammasome might be a potential immune intervention against severe COVID-19 disease.

## Materials and Methods

### Regents, cells and virus

The primary antibodies and chemicals used in the present study are listed in Table S1. The human monocytic cell line THP-1 (ATCC TIB-202; RRID: CVCL_0006) was used to model macrophages. Briefly, THP-1 cells were cultured in RPMI-1640 medium (Gibco) supplemented with 10% fetal bovine serum (FBS) (Gibco) and kept in a humidified 5% CO_2_ incubator at 37°C. The SARS-CoV-2 strain was kindly provided by Guangdong Provincial Center for Disease Control and Prevention, Guangdong Province of China and was described in our previous studies.^43^,^44^ The virus was propagated and titrated in Vero E6 cells, which were cultured in Dulbecco's Modified Eagle medium (DMEM) supplemented with 10% FBS. The viral sequence is accessible in the China National Microbiology Data Center (Accession No. NMDCN0000HUI).

### Stable hACE2-overexpressing cell lines

THP-1 cells overexpressing *hACE2* were generated by lentiviral transduction. Briefly, the lentivirus was made by co-transfection of lentiviral transfer vector carrying *hACE2* coding sequences (pLVX-EF1a-Puro) and packaging plasmids pMD2G (Cat # 12259; Addgene) and psPAX2 (Cat # 12260; Addgene) into HEK293T cells using Lipofectamine 3000 (Thermo Fisher Scientific). The lentivirus-containing supernatant was collected and pooled at 72 h post-transfection. THP-1 cells were transduced by the lentivirus at 1:1 dilution of culture medium and lentivirus-containing supernatant in the presence of 8 μg/mL polybrene (Millipore). Stable cells overexpressing *hACE2* were selected and maintained in growth medium with 1 μg/mL puromycin (InvivoGen). Then, stable THP-1 were differentiated to THP-1-derived macrophages by 0.08 μM of phorbol 12-myrisate 13-acetate (PMA) (Sigma-Aldrich) for 48 h.

### Infection and treatment of THP-1-derived macrophages

Lentiviral vector-mediated *in vitro* gene delivery was performed as previously described.^45^ Briefly, *hACE2*-overexpressing THP-1 cells were cultured with medium containing 8 μg/mL polybrene (Millipore) and 5 × 10^5^ infectious units of lentiviruses containing scrambled shRNA or *NLRP3*-specific shRNAs (target sequence for human: 5’-CCGTAAGAAGTACAGAAAGTA-3’). At 48 h post infection, cells were infected with SARS-CoV-2 as indicated. In brief, THP-1 cells overexpressing *hACE2* were primed with 250 ng/mL Pam3Cys (InvivoGen) for 4 h and then incubated with SARS-CoV-2 at a multiplicity of infection (MOI) of 1 for 24 h. No infection (Mock) and nigericin (20 μM; Millipore) were used as a negative control and a positive control for NLRP3 activation, respectively. The NLRP3 inflammasome inhibitor MCC950 (15 μM; Selleck) was administered 1 h later after viral infection.

### Animal models

Male *Nlrp3*-KO mice (10-12 weeks old) as described in our previous study^46^ and male C57BL/6J mice with matched ages were maintained in the Experimental Animal Core Facility of the Kunming Institute of Zoology (KIZ), Chinese Academy of Sciences (CAS). 8-12 weeks old male hACE2 transgenic mice were purchased from the Shanghai Model Organisms.

For AAV-hACE2 infection in mice, C57BL/6J (*n* = 5) and *Nlrp3*-KO (*n* = 5) mice were intranasally incubated with *hACE2*-expressing AAV (AAV-hACE2) (PackGene Biotech, Guangzhou) at a total of 40 μl containing 4 × 10^12^ viral genome copies (GC) (20 μl per nostril). All mice were monitored daily until SARS-CoV-2 infection at 14 d post AAV-hACE2 infection. For treatment of NLRP3 inflammasome inhibitor MCC950, hACE2 transgenic mice (*n* = 12) were assigned into two groups (drug group: *n* = 6; control group, *n* = 6). The drug group received an intraperitoneal injection of the NLRP3 inflammasome inhibitor MCC950 (Selleck) at a dose of 10 mg/kg 1 h before SARS-CoV-2 infection, and thereafter a daily injection with the same dose. The control group received the same volume of 1 × PBS intraperitoneally and followed the same procedure as for the drug group.

After anesthetization with isoflurane (RWD Life Science), mice were intranasally infected with a total of 20 μl containing 1 × 10^5^ Median Tissue Culture Infectious Dose (TCID_50_) of SARS-CoV-2. Body temperature and weight of all mice were recorded daily until sacrifice at 4 d post infection. Blood samples were collected for routine blood tests before infection and just before sacrifice. Tissue samples were collected from animals after euthanasia and stored in -80°C freezer (for quantification of mRNA and protein levels) or in 4% paraformaldehyde (PFA; for histological analysis and immunofluorescence) until use.

### Quantitative real-time PCR (qRT-PCR)

Total RNA was extracted from homogenized mouse tissues using a TRIzol reagent (Thermo Fisher Scientific). SARS-CoV-2 RNAs were detected by one-step RT-PCR using a THUNDERBIRD Probe One-Step qRT-PCR (TOYOBO, Japan) following the manufacturer's protocols. The primers targeting the N protein described in our previous study^43^ were used, including 5’-GGGGAACTTCTCCTGCTAGAAT-3’, 5’-CAGACATTTTGCTCTCAAGCTG-3’, probe 5’-FAM-TTGCTGCTGCTTGACAGATT-TRMRA-3’. Serial dilutions of the SARS-CoV-2 RNA reference standard (National Institute of Metrology, China) were used in each run, in parallel to calculate copy numbers in each sample. To detect RNA transcript of inflammatory mediators, the target transcripts were determined by quantitative PCR using qScriptTM One-Step qRT-PCR kit (Quanta Biosciences, #95057-050) on CFX96 real-time PCR system (Bio-Rad). The primer sequences are listed in Table S2.

### Histological analysis

We followed the same histological approach as described in our previous studies.^43^,^44^ In brief, lung tissues form SARS-CoV-2-infected mice were fixed in 4% PFA (Biosharp) for at least 7 d, and then were paraffin embedded (Thermo Fisher Scientific) and cut into 3-μm sections following the standard procedure (RWD Life Science). The tissue slides were stained with H&E (Solarbio) and imaged by the Leica DM 6B light microscopy. The pathological score was evaluated based on the degree of lung tissue lesions including alveolar septal thickening, hemorrhage, inflammatory cell infiltration, and consolidation. The semiquantitative assessment was performed as described previously.^47^

### Immunofluorescence

Immunofluorescence was performed as described in our previous studies.^43^,^45^,^48^ Briefly, the sections were deparaffinized in xylene and rehydrated through a graded ethanol series. For antigen retrieval, sections immersed in saline sodium citrate buffer were microwave heated for 10 min three times. After cooled to room temperature, the sections were washed by 1×phosphate-buffered saline (PBS; Solarbio), and blocked with 5% donkey serum (Millipore) in 1×PBST (0.3% Triton-X 100 in PBS) at 37°C for 60 min. The commercial antibodies listed in Table S1 were diluted in 3% donkey serum) in 1×PBST (0.2% Triton-X 100) and incubated overnight at 4°C. The sections were then washed, and immunoreactivity was detected using Donkey anti-Rabbit IgG Highly Cross-Adsorbed Secondary Antibody, Alexa Fluor Plus 488 or Donkey anti-Mouse IgG Highly Cross-Adsorbed Secondary Antibody, Alexa Fluor Plus 555 (1:500; Thermo Fisher Scientific) for 1 h at room temperature. The sections were counterstained with 5 μg/mL 4’,6-diamidino-2-phenylindole (DAPI; Thermo Fisher Scientific) for 10 min at room temperature and washed with 1×PBST (0.2% Triton-X 100) three times. Slides were visualized using an Olympus FluoView 1000 confocal microscope (Olympus).

### Immunoblotting

Immunoblotting was performed as previously described.^45^ Briefly, target cells were lysed in 1% NP40 buffer (Beyotime) with protease inhibitor cocktail (MedChemExpress). Proteins were separated by SDS-PAGE and transferred to PVDF membrane by semi-dry transfer at 25 V for 30 min. The membrane was blocked in 5% skim milk in PBST for 1 h and incubated overnight with commercial primary antibody (Table S1) in 5% bovine serum albumin (BSA) at 4°C. The membrane was incubated with anti-mouse HRP-conjugated secondary antibodies in 5% milk and bands were developed with Chemi-Doc XRS imaging (Bio-Rad).

### Active caspase-1 activity and LDH release

THP-1 derived macrophages stably overexpressing hACE2 (4 × 10^5^ cells) were plated on 24-well plates in RPMI-1640 medium supplemented with 10% FBS and incubated overnight. Cells were then infected with SARS-CoV-2 at an MOI of 1 in RPMI-1640 culture medium without Phenol Red for 1 h to allow virus adsorption. After virus inoculation, fresh RPMI-1640 medium supplemented with 2% FBS with or without 15 μM MCC950 were added for 24 h. To determine caspase-1 activity, culture supernatants were collected and incubated with the luciferin WEHD-substrate in the Caspase-Glo 1 Assay (Promega). After an incubation for 1 h at room temperature, luminescence was detected using the SpectraMax i3 system (Molecular Devices). LDH release was measured in the supernatants using the CytoTox 96 Non-Radioactive Cytotoxicity Assay (Promega) following the manufacturer's instructions.

### Enzyme-linked immunosorbent assay (ELISA) for IL-1β and IL-18

The levels of IL-1β and IL-18 in the supernatants from cultured cells were measured by commercial ELISA assay (R&D Systems) following the manufacturer's instructions. ELISA values were determined using the SpectraMax i3 system (Molecular Devices).

### Ethics statement

All experiments with live SARS-CoV-2 were conducted in the animal biosafety level 3 (ABLS3) facility in the KIZ, CAS. The Institutional Animal Care and Use Committee of KIZ, CAS, approved all experimental procedures and protocols used in this study (Approval No. SMKX-2021-03-015-02).

### Statistical analysis

All appropriate data were analyzed using GraphPad Prism 8 (GraphPad Software Inc.). All hypothesis tests were performed as two-tailed tests. Specific statistical analysis methods are described in the figure legends where results are presented. Values were considered statistically significant for *p* values < 0.05.

### Funding sources

This work was supported by grants from the Bureau of Frontier Sciences and Education, CAS (grant no. QYZDJ-SSW-SMC005 to Y.G.Y.), the key project of the CAS “Light of West China” Program (to D.Y.) and Yunnan Province (202001AS070023 to D.Y.).

### Role of funding source

The sponsors of funding had no role in study design, data collection, data analyses, interpretation, or writing of report.

## Results

### Inhibition of the NLRP3 inflammasome attenuates immune overactivation and viral replication in SARS-CoV-2 infected immune cells

**Figure 1:**
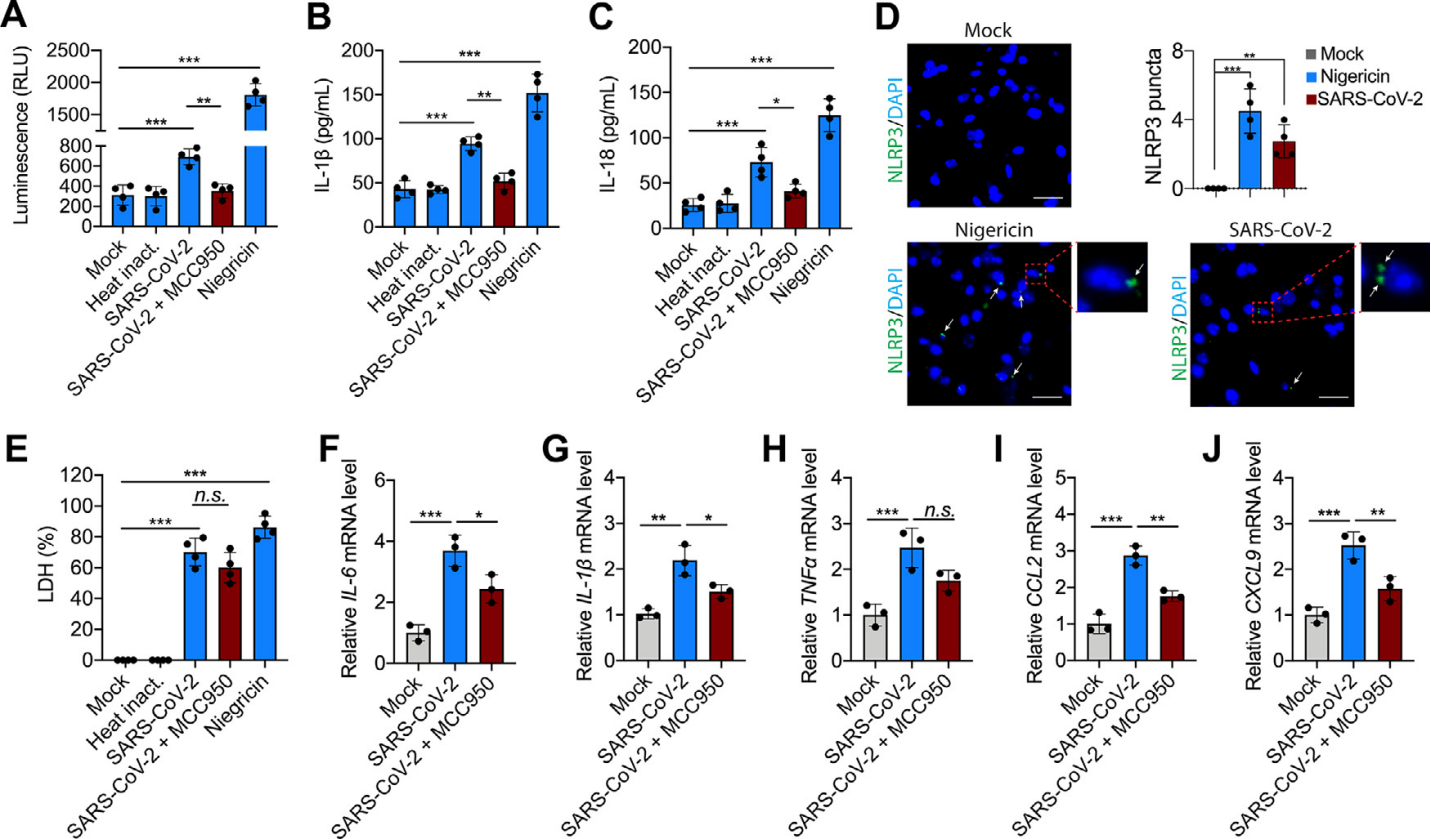
SARS-CoV-2 infection induces NLRP3 inflammasome activation in cultured immune cells. Human THP-1 derived macrophages overexpressing human *ACE2* (*hACE2*) were primed with 250 ng/mL Pam3Cys for 3 h and infected with SARS-CoV-2 at an MOI of 1 for 24 h. Mock (uninfected) was used a negative infection control and nigericin as a positive control for NLRP3 activation. The NLRP3 inhibitor MCC950 (15 μM) was added 1 h after SARS-CoV-2 infection and maintained for 24 h. The SARS-CoV-2 was also inactivated by 56°C for 30 min (Heat inact.) as another control in the assay. (A) Caspase-1 activity was measured in the culture supernatants using the Caspase-Glo 1 assay. RLU – relative luminescence units. (B and C) The protein levels of IL-1β (B) and IL-18 (C) in the culture supernatants determined by ELISA. Values in (A-C) are presented as Mean ± SD; *n* = 4; *p < 0.05, **p < 0.01, and ***p < 0.001, one-way ANOVA with Bonferroni's post *hoc* test. (D) The macrophages were stained with anti-NLRP3 (green) for determining NLRP3 puncta (white arrow). Scale bar, 20 μm. Number of the NLRP3 puncta was quantified from the average of two fields each well. Mean ± SD; *n* = 4; **p < 0.01 and ***p < 0.001, one-way ANOVA with Bonferroni's *post hoc* test. (E) LDH release in the culture supernatants. Cells treated by 8% Triton X-100 had 100% cell death and were used as the normalization standard. Mean ± SD; *n* = 4; *n.s.*, not significant, ***p < 0.001, one-way ANOVA with Bonferroni's post *hoc* test. (F-J) Quantification of mRNA levels of pro-inflammatory cytokines *IL-6* (F), *IL-1β* (G), *TNF-α* (H), *CCL2* (I), and *CXCL9* (J) in the cell lysates via the quantitative real-time PCR (qRT-PCR). Mean ± SD; *n* = 3; *n.s.*, not significant, *p < 0.05, **p < 0.01, and ***p < 0.001, one-way ANOVA with Bonferroni's *post hoc* test.

The NLRP3 inflammasome, which functions as an important part of the innate immune system, has been shown to be associated with COVID-19 severity in patients.^34^ We were stimulated to investigate whether and how the inflammatory response and SARS-CoV-2 replication were affected by modulating NLRP3 inflammasome activation using an inhibitory small molecule and lentivirus-mediated knockdown. We first developed immune THP-1 derived macrophages stably overexpressing the hACE2 receptor to make it susceptible to SARS-CoV-2 entry (Fig. S1A). By using this susceptible cell line, we found that caspase-1 was activated by SARS-CoV-2 infection according to the Caspase-Glo 1 luminescent assay that was used to detect caspase-1 activity in the media (Figure. 1A). Activated caspase-1 cleaves pro-IL-1β and pro-IL-18 into mature IL-1β and IL-18, respectively, which are released from cells. We have shown SARS-CoV-2 triggered the production of mature IL-1β (Figure. 1B) and IL-18 (Figure. 1C) in the culture supernatants. We also observed the formation of NLRP3 puncta in macrophages with SARS-CoV-2 infection or with the positive control Nigericin treatment (Figure. 1D), which is a toxin to trigger the NLRP3 inflammasome.^49^ Heat-inactivated virus failed to trigger caspase-1 activation and related cytokine production (Figure. 1A-C), demonstrating that caspase-1 activation and related cytokine production exclusively required viable SARS-CoV-2. Notably, MCC950, a potent and selective inhibitor of the NLRP3 inflammasome,^42^ showed an inhibitory effect on SARS-CoV-2-induced caspase-1 activation and IL-1β and IL-18 productions (Figure. 1A-C).

The inflammatory overactivation of COVID-19 was generally associated with infected cell death, such as pyroptosis, and disease severity.^14^,^16^,^18^,^25^,^36^ SARS-CoV-2 triggered lactate dehydrogenase (LDH) release (Figure. 1E), a marker of the pyroptosis, in the macrophages; whereas MCC950 had no such an effect on LDH release, suggesting that NLRP3 does not participate in SARS-CoV-2-induced cell death. In fact, the NLRP3 inflammasome plays an important role in host inflammatory status through IL-1β- and IL-18-mediated regulatory mechanisms.^27^ We performed quantitative real-time PCR (qRT-PCR) for representative COVID-19 related host inflammatory cytokine genes and found that a cytokine panel (including IL-6, IL-1β, TNF-α, CCL2, and CXCL9) was induced by SARS-CoV-2 infection. Such an induction of cytokines could be significantly inhibited by MCC950 treatment (Figure. 1F-J).

We achieved a successful lentivirus-mediated *NLRP3* knockdown in the THP-1 derived macrophages stably overexpressing *hACE2*, as confirmed by the decreased *NLRP3* mRNA transcript levels (Fig. S1B). Also, *NLRP3* knockdown caused a reduction of caspase-1 activity (Fig. S1C) and a decrease of mature IL-1β (Fig. S1D) and IL-18 (Fig. S1E); although LDH release was not significantly affected by *NLRP3* knockdown (Fig. S1F). Collectively, these data demonstrated that the NLRP3 inflammasome is engaged in SARS-CoV-2 related innate immunity in immune cells.

### *Nlrp3* deficiency ameliorates SARS-CoV-2 induced lung pathology in a mouse model

Since the NLRP3 inflammasome was involved in SARS-CoV-2 related immune response, we further investigated the role of the NLRP3 inflammasome in the immunopathology of COVID-19 disease by using *Nlrp3* knockout (*Nlrp3*-KO) mice. The wild-type C57BL/6J mice were used as control. To make the mice susceptible to SARS-CoV-2 infection, they were made to overexpress hACE2 protein, the receptor for SARS-CoV-2 entry, by intranasal infection of adenovirus-associated virus carrying the *hACE2* gene (AAV-hACE2) in C57BL/6J wild type and *Nlrp3*-KO mice. Successful expression of hACE2 in lung tissue in these animals was confirmed by immunoblot using human ACE2 antibody (Fig. S2A). We did not observe significant changes regarding body temperature and body weight within 4 consecutive days post SARS-CoV-2 infection (Fig. S2B). It should be pointed out the body weight of some mice experienced unregular change but the exact reason remained to be explored. Routine blood tests showed an increased level of white blood cells (WBC) (Fig. S2C) and monocytes (Fig. S2D) during the viral infection, similar to the pattern observed in tree shrews infected by SARS-CoV-2 in our previous study.^43^ However, there was a reduced enhancement of WBC (Fig. S2C) and monocytes (Fig. S2D) before viral infection and at 4 dpi in *Nlrp3*-KO mice compared to C57BL/6J mice. No such change was found in granulocytes (Fig. S2E) and lymphocytes (Fig. S2F), suggesting a potential cell-type specific reaction upon SARS-CoV-2 infection.

**Figure 2:**
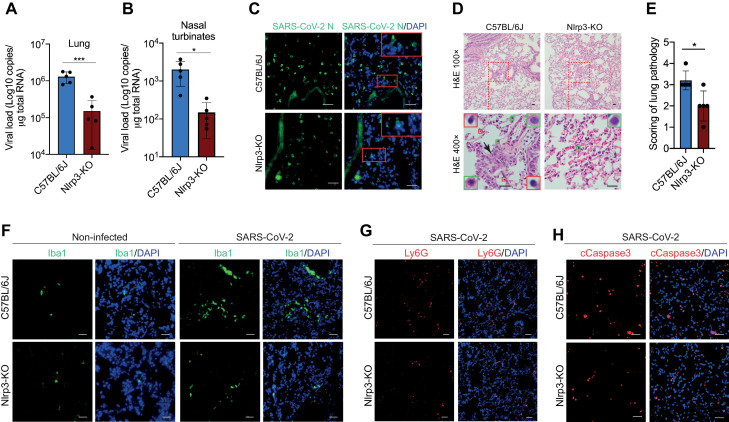
*Nlrp3* deficiency alleviates COVID-19 like pathology in the mouse model. (A and B) Quantification of viral loads in tissues of SARS-CoV-2 infected animals. C57BL/6J and *Nlrp3*-KO mice were intranasally incubated with hACE2-expressing AAV (AAV-hACE2) at a total of 40 μl containing 4 × 10^12^ viral genome copies (GC) (20 μl per nostril). All mice were monitored daily until SARS-CoV-2 infection. Viral loads in lungs (A) and nasal turbinates (B) at 4 dpi of C57BL/6J and *Nlrp3*-KO mice pre-injected with AAV-hACE2 2 weeks before SARS-CoV-2 nasal infection were quantified by using quantitative real-time PCR. Mean ± SD; *n* = 5; *p < 0.05 and ***p < 0.001, Student's *t*-test. (C) Immunofluorescence of anti-SARS-CoV-2 N protein in lung tissues from C57BL/6J and *Nlrp3*-KO mice at 4 dpi. Scale bar, 30 μm. (D and E) Histopathology of lung tissues from SARS-CoV-2 infected C57BL/6J and *Nlrp3*-KO mice at 4 dpi (D). Histopathological observations indicated the presence of moderate interstitial pneumonia with thickened alveolar septa (black arrow) and infiltration of lymphocytes (red frames; magnification). The swollen and degenerative mononuclear cells (green frames; magnification) are scattered within the alveolar cavities. Scale bar, 50 μm. The pathology severity was scored (E). Mean ± SD; *n* = 5; *p < 0.05, Student's *t*-test. (F-H) Immunofluorescence of macrophage marker Iba1 (F), neutrophil marker Ly6G (G), and cell death marker cleaved caspase-3 (cCaspase-3) (H) in lung tissues from non-infected or SARS-CoV-2 infected C57BL/6J and *Nlrp3*-KO mice at 4 dpi. Scale bar, 30 μm.

Quantification of viral loads in *Nlrp3*-KO mice showed 8- and 10-fold reduction of viral load in the lungs (Figure. 2A) and nasal turbinates (Figure. 2B), respectively, compared to C57BL/6J mice at 4 dpi. We used immunofluorescence on lung tissue to detect SARS-CoV-2 nucleoprotein (N) and found a consistent reduction of viral protein in Nlrp3-KO mice relative to that of infected C57BL/6J mice (Figure. 2C). Histological analysis showed that lung tissue of SARS-CoV-2 infected *Nlrp3*-KO mice had fewer signs of interstitial pneumonia, characterized by thinner alveolar septa accompanied by a decrease in the number of inflammatory cells, and lowered accumulation of inflammatory cells in partial alveolar cavities, compared to those of infected C57BL/6J mice (Figure. 2D). The infected C57BL/6J mice had more inflammatory cells (including lymphocytes, macrophages, and neutrophils) in the alveolar tissue than in the infected *Nlrp3*-KO mice (Figure. 2D). A similar pattern was found for the scored lung pathologies (Figure. 2E). To determine the infiltration of specific inflammatory cells, we performed immunofluorescence to identify Iba1^+^ macrophages and Ly6G^+^ neutrophils. Compared to the lungs of SARS-CoV-2 infected C57BL/6J mice, fewer macrophages and neutrophils were found in the lungs of SARS-CoV-2 infected *Nlrp3*-KO mice. Compared to non-infection, the diffused infiltrations of Iba1^+^ macrophages induced by SARS-CoV-2 infection into the alveolar cavities were reduced by *Nlrp3* deficiency (Figure. 2F). Meanwhile, some Ly6G^+^ neutrophils focally aggregated in the alveolar septa (Figure. 2G). SARS-CoV-2 infection also induced cellular inflammatory response and cell death, as revealed by the decreased staining of cell death marker cleaved caspase-3 (cCaspase-3) in the lungs of SARS-CoV-2 infected *Nlrp3*-KO mice relative to the controls (Figure. 2H). Collectively, these *in vivo* results suggest that the NLRP3 inflammasome plays an important role in SARS-CoV-2 induced lung pathology.

### Inactivation of NLRP3 inflammasome relieves COVID-19 related inflammatory overactivation

**Figure 3:**
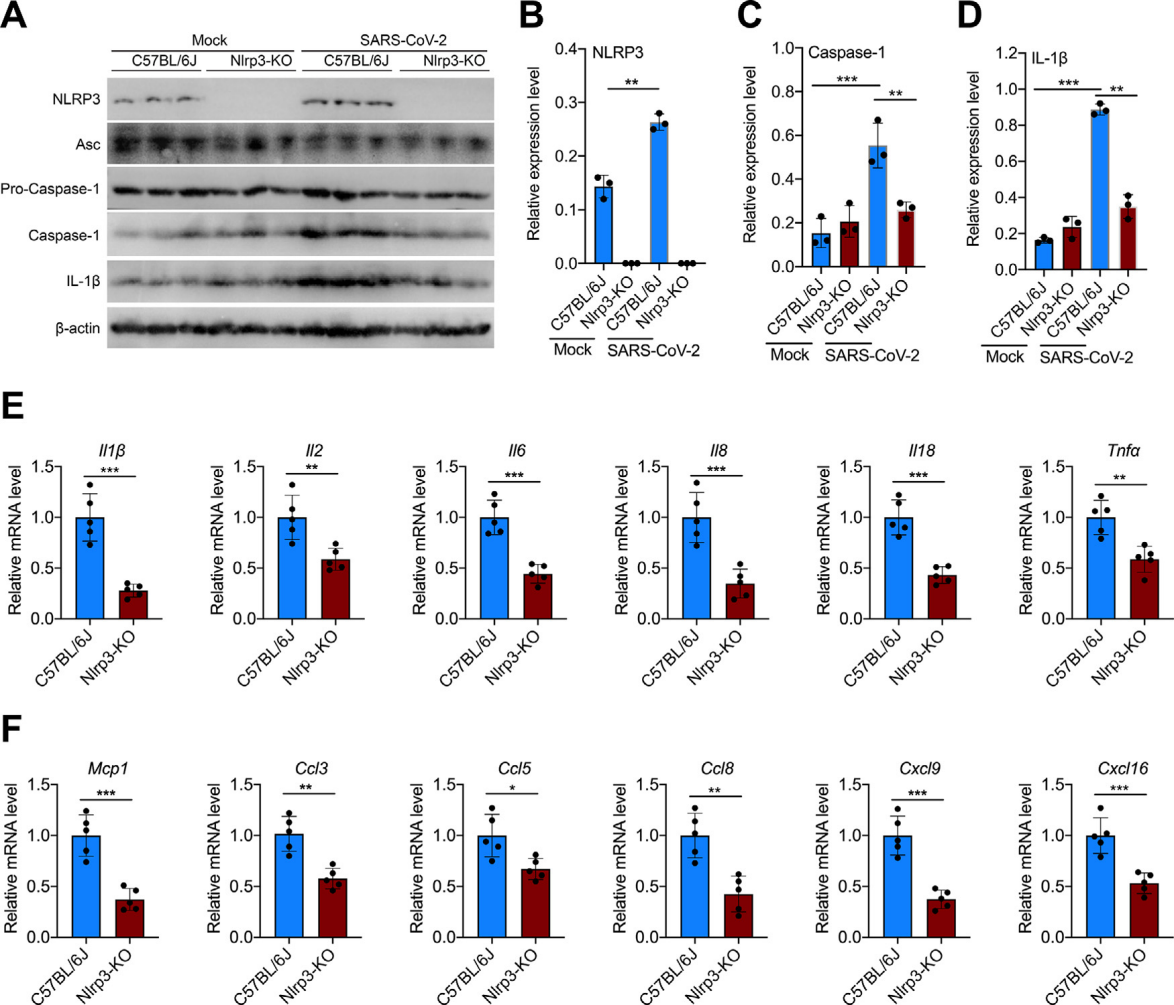
Decrease of COVID-19 related inflammatory response in *Nlrp3*-KO mice. (A) Immunoblot of NLRP3 inflammasome molecules including NLRP3, Asc, Pro-caspase-1, caspase-1, and IL-1β in lung tissues of C57BL/6J and *Nlrp3*-KO mice without (Mock) or with SARS-CoV-2 infection at 4 dpi. (B-D) Quantification of protein expression levels of NLRP3 (B), caspase-1 (C), and IL-1β (D) relative to β-actin in (A). Mean ± SD; *n* = 3; **p < 0.01, ***p < 0.001, one-way ANOVA with Bonferroni's post *hoc* test. (E) Relative mRNA levels of pro-inflammatory cytokines *Il1β, Il2, Il6, Il8, Il18* and *Tnfα* in lung tissues of SARS-CoV-2 infected C57BL/6J and *Nlrp3*-KO mice at 4 dpi. *β-actin* was used as a control for normalization during the qRT-PCR. Mean ± SD; *n* = 5; **p < 0.01, ***p < 0.001, Student's *t*-test. (F) Relative mRNA levels of pro-inflammatory chemokines *Mcp1, Ccl3, Ccl5, Ccl8, Cxcl9* and *Cxcl16* in lung tissues of SARS-CoV-2 infected C57BL/6J and *Nlrp3*-KO mice at 4 dpi. Procedure and statistical analysis were same to (E).

Since *Nlrp3* deficiency alleviated COVID-19 like pathology in lungs, we were stimulated to study the underlying mechanism. We first checked whether SARS-CoV-2 stimulated NLRP3 inflammasome activation of the lung in C57BL/6J and *Nlrp3*-KO mice. Activation of NLRP3 inflammasome resulted in the production of enzymatically active caspase-1 and mature IL-1β.^28^ Immunoblot assay showed that SARS-CoV-2 infection not only induced NLRP3 expression (Figure. 3A-B), but also strongly stimulated the NLRP3 inflammasome in C57BL/6J mice, as shown by the increased levels of caspase-1 and IL-1β in the lung tissues (Fig. 3A, C-D). However, SARS-CoV-2 infection failed to induce caspase-1 and IL-1β production in the lung tissues of *Nlrp3*-KO mice (Figure. 3A, C-D).

NLRP3 inflammasome activation primed and stimulated host inflammatory response,^27^ and the inflammatory cytokines (including IL-1β, IL-2, IL-6, IL-8, IL-18, and TNF-α) have been identified to be related to disease severity in COVID-19 patients.^26^ We therefore examined the expression of these inflammatory cytokines in SARS-CoV-2 infected lung tissues and found that the COVID-19 related inflammatory cytokines were significantly decreased in the infected lung tissues of *Nlrp3*-KO mice compared to C57BL/6J mice (Figure. 3E). Inflammatory chemokines, such as MCP-1, CCL3, CCL5, CCL8, CXCL9, and CXCL16, are also important in the SARS-CoV-2 induced inflammatory response cascade.^26^ In the lung tissues of *Nlrp3*-KO mice, the mRNA levels of these COVID-19 related chemokines were reduced relative to C57BL/6J mice upon SARS-CoV-2 infection (Figure. 3F). Collectively, these *in vivo* data demonstrated that Nlrp3 deficiency disables SARS-CoV-2 induced inflammasome activation and thus inhibits inflammatory responses to alleviate COVID-19 like immunopathology in lungs.

### MCC950 inhibits SARS-CoV-2 induced immune overactivation to relieve COVID-19 like pathology in hACE2 transgenic mice

**Figure 4:**
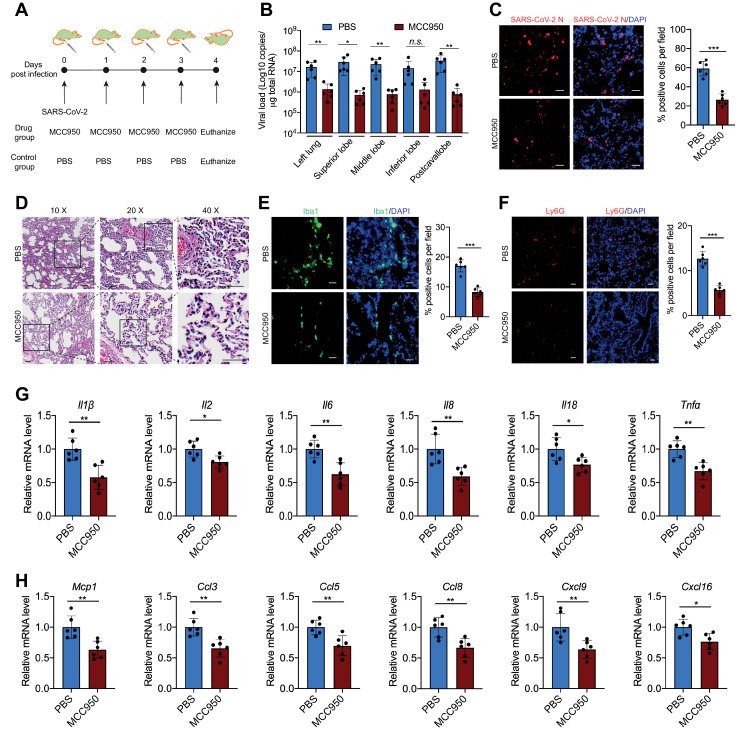
Targeting NLRP3 inflammasome by MCC950 treatment ameliorates immune overactivation and COVID-19 like pathology in lung tissues of hACE2 transgenic mice. (A) Diagram of animal experiments. hACE2 transgenic mice were intranasally infected with 1 × 10^5^ TCID_50_ of SARS-CoV-2 and followed by daily intraperitoneal administration of 10 mg/kg MCC950 (MCC950-treated group) or equal amount of 1 x PBS solution (PBS-treated group). All mice were euthanized at 4 dpi. (B) Viral loads in the five lung lobes of the MCC950-treated and PBS-treated mice at 4 dpi. Mean ± SD; *n* = 6; *n.s.*, not significant, *p < 0.05, **p < 0.01, Student's *t*-test. (C) Immunofluorescence and quantification of anti-SARS-CoV-2 N protein in lung sections of hACE2 transgenic mice at 4 dpi. Scale bar, 30 μm. Mean ± SD; *n* = 6; ***p < 0.001, Student's *t*-test. (D) Histopathology of lungs from SARS-CoV-2 infected hACE2 transgenic mice at 4 dpi. Scale bar, 50 μm. (E and F) Immunofluorescence and quantification of macrophage marker Iba1 (E) and neutrophil marker Ly6G (F) in lung sections at 4 dpi. Scale bar, 30 μm. Mean ± SD; *n* = 6; ***p < 0.001, Student's *t*-test. (G) Relative mRNA levels of pro-inflammatory cytokines *Il1β, Il2, Il6, Il8, Il18* and *Tnfα* in lung tissues at 4 dpi. *β-actin* was used for normalization in qRT-PCR. Mean ± SD; *n* = 6; *p < 0.05, **p < 0.01, Student's *t*-test. (H) Relative mRNA levels of inflammatory chemokines *Mcp1, Ccl3, Ccl5, Ccl8, Cxcl9* and *Cxcl16* in lung tissues at 4 dpi. Mean ± SD; *n* = 6; *p < 0.05, **p < 0.01, Student's *t*-test.

We next investigated the potential of NLRP3 specific inhibitor MCC950 as a potential immune intervention against COVID-19 disease using the hACE2 transgenic (hACE2-tg) mouse model (Figure. 4A). SARS-CoV-2 infection significantly triggered the NLRP3 inflammasome activation in hACE2-tg mice (Fig. S3A-B) and treatment with MCC950 significantly suppressed the NLRP3 inflammasome activation triggered by SARS-CoV-2 infection in lung tissues of hACE2-tg mice, as shown by the reduction of mature caspase-1 expression at 4 dpi (Fig. S3A-B). Also, SARS-CoV-2 load levels in left lung, superior lobe, middle lobe, and postcaval lobe were reduced in MCC950-treated group compared to the PBS-treated group at 4 dpi (Figure. 4B). The reduction of SARS-CoV-2 nucleoprotein (N) was observed in the lung sections of MCC950-treated mice compared to those of PBS-treated mice (Figure. 4C). Histological analysis showed that lung tissues of MCC950-treated mice had reduced immunopathology, as shown by fewer signs of interstitial pneumonia and a decreased accumulation of inflammatory cells compared to the PBS-treated mice (Figure. 4D). To further determine the infiltration of specific inflammatory cells, we performed immunofluorescence to identify Iba1^+^ macrophages (Figure. 4E) and Ly6G^+^ neutrophils (Figure. 4F). Compared to the lungs of PBS-treated mice, fewer Iba1^+^ macrophages and Ly6G^+^ neutrophils were observed in the lungs of MCC950-treated mice (Figure. 4 E-F).

We next used a specific panel of COVID-19 related inflammatory cytokines and chemokines to look at the samples from the MCC950-treated hACE2-tg mice. The mRNA levels of inflammatory cytokines were significantly reduced in the lung tissues of the MCC950-treated mice compared to PBS-treated mice (Figure. 4G). Similarly, the COVID-19 related chemokines were also decreased in MCC950-treated mice compared to PBS-treated mice (Figure. 4H). Taken together, our results demonstrated that specific inhibition of the NLRP3 inflammasome suppresses SARS-CoV-2 induced immune overactivation in the lungs and alleviates COVID-19 like immunopathology, and that targeting the NLRP3 inflammasome might be a potential immune intervention of severe COVID-19 disease.

## Discussion

Overactivated or dysregulated Inflammation is an important pathological element in many human diseases, such as those caused by viral infection and neurodegeneration.^50^ The NLRP3 inflammasome, as the central innate immune sensor priming systematic host inflammatory response to pathogenic stimulation, has been extensively studied in many human diseases.^27^,^30^,^31^ As a result of these studies, the NLRP3 inflammasome has been proposed as being a promising target for drug development. Most recently, it has been reported that the NLRP3 inflammasome is activated in response to SARS-CoV-2 infection.^32^,^33^ Moreover, NLRP3 inflammasome is associated with COVID-19 severity in patients.^34^, ^35^, ^36^, ^37^ In addition to direct lung damage, SARS-CoV-2 infection induced a systematic cytokine storm, a status of host immune overactivation.^12^,^17^,^19^,^25^ The development of a cytokine storm has been considered as a pathological process responsible for severe COVID-19 disease progression.^14^,^15^,^20^,^22^,^26^,^34^ It has been confirmed that NLRP3 inflammasome activation in response to SARS-CoV-2 replication is closely associated with COVID-19 related cytokine storm in patients with severe disease.^38^,^51^ For example, SARS-CoV-2 infected blood monocytes to activate the NLRP3 inflammasome, and lead to the formation of a COVID-19 related cytokine storm.^41^ The high prevalence of COVID-19 cases in the elderly is likely due to the compromised ability to tightly regulate the host immune response. NLRP3 inflammasome was over-stimulated, as evidenced by the increased lethality in SARS-CoV-2 infected elderly patients.^40^ These clinic observations consistently suggested that the NLRP3 inflammasome actively participated in severe COVID-19 disease. Compared to the available knowledge, our current study was focused on mostly *in vivo* study to uncover the importance of NLRP3 inflammasome in COVID-19 lung immunopathology. Although it would be worthwhile to examine the role of NLRP3 in individual immune cells by cell-specific *Nlrp3* deficiency, the present study served as the first step using systematically genetic knockout and/or pharmacological inhibition of NLRP3 inflammasome to characterize the role of NLRP3 in the pathogenesis of COVID-19, and we found the potential of targeting NLRP3 inflammasome as immune intervention of COVID-19 disease.

Excessive inflammatory response stimulated by SARS-CoV-2 causes both mild and severe immunopathology in lungs,^20^ and therefore treating excessive or dysregulated inflammation might be an important approach for curing COVD-19. However, there is so far no fully accepted drug available to combat COVID-19 in patients, albeit there were reports for the controversial efficacy of remdesivir, chloroquine and hydroxychloroquine for the treatment of COVID-19.^52^ Our present study showed that the specific NLRP3 inhibitor could effectively reduce the COVID-19 related inflammatory cytokine storm and alleviate lung immunopathology in the mouse model. Thus, our study paves the way to exploit the potential of the NLRP3 inflammasome as a target for drug therapy, especially in the treatment of severe COVID-19 disease.

Cell death including pyroptosis is another pathological event induced by SARS-CoV-2 infection. SARS-CoV-2 Spike protein interacted with Toll-like receptor 4 (TLR4) and activated TLR4-mediated host innate immunity,^53^ and a TLR4 receptor inhibitor TAK-242 significantly inhibited the Spike-induced cell pyroptosis.^54^ Because NLRP3 inhibitor MCC950 treatment or NLRP3 knockdown did not affect live SARS-CoV-2-induced macrophage cell death determined by LDH release (Figure. 1E and Fig. S1F), this result suggests that NLRP3-independent responses operate for induction of a lytic form of cell death induced by SARS-CoV-2. Recently, another independent study also reported such an NLRP3-independent live SARS-CoV-2-induced cell death in monocytes.^34^ These results suggest a complicated regulation of SARS-CoV-2 induced cell damage. In addition, there are at least two possible speculations for novel mechanism of the role of NLRP3 in affecting the SARS-CoV-2 replication. One is that NLRP3 directly participates in the formation of SARS-CoV-2 replication complex composed of viral proteins, thereby facilitating viral replication; however, this has not been validated so far. The other one is that NLRP3 inflammasome activation produces a virus-preferred immune environment which contributes to SARS-CoV-2 replication. Future study should be performed to confirm these speculations.

There are several limitations of this study. First, at the time of our manuscript preparation, Pan et al.^39^ found that SARS-CoV-2 N protein facilitates the binding of NLRP3 with ASC and promotes NLRP3 inflammasome assembly to contribute to the maturation of pro-inflammatory cytokines. This elaborate mechanism, together with our *in vivo* study, jointly supported the promise of the NLRP3 inflammasome as a valid target in treating COVID-19 related immune activation although we did not perform an assay to verify the binding of NLPR3 with ASC in our system. In addition, SARS-CoV-2 infection induced NLRP3 expression (Figure. 3A-B) and such an induction was also observed in other types of virus infection.^55^,^56^ Thus, both these events are likely to jointly facilitate the activation of NLRP3 inflammasome signaling, as reflected by the increase of pro-caspase-1 cleavage and pro-inflammatory cytokines. Second, we used the *Nlrp3*-KO mice and hACE2-tg mice to display the role of the NLRP3 inflammasome in SARS-CoV-2 infection and development of COVID-19 disease, independent evaluation of targeting the NLRP3 inflammasome in a non-human primate such as *rhesus macaque* is needed. Third, although we found that targeting the NLRP3 inflammasome using MCC950 effectively alleviates SARS-CoV-2 induced immune activation, the efficacy of MCC950 treatment or a combination of the NLRP3 inhibitor with other important immune inhibitors should be tested in a clinical trial. Fourth, although the differentiated role of NLRP3 inflammasome in specific cell types such as macrophage, monocytes, and dendritic cells in COVID-19 patients has been preliminarily shown by single cell RNA-seq,^34^,^57^,^58^ it would be rewarding to investigate the role of NLRP3 inflammasome and related signaling pathways in individual immune cells in COVID-19 context.

In conclusion, our current study demonstrated that targeting NLRP3 inflammasome by MCC950 is a potential treatment for COVID-19 related immunopathology in mouse models. Next, this concept should be further tested in other models such as non-human primate and tree shrew infection models^43^,^44^,^59^ before initiating a clinical trial. In addition, a combination of NLRP3 inhibitors with other immunomodulators could be considered as promising therapeutics for treating COVID-19 patients with specific conditions, for example severe COVID-19 patients with prominent cytokine storm.

## Contributors

All authors read and approved the final version of the manuscript, and ensured it is the case. JZ, YGY, and MHL conceived of the research, designed the study. JZ and YGY wrote the manuscript. JZ, XX, XLF, LX, JBH, DDY, QCZ, XL and GM performed the experiments and analyzed data. QJL coordinated the supply of the *Nlrp3*-KO mice. All authors commented on the manuscript.

## Declaration of interests

The authors declare no competing interests.

## Appendix Supplementary materials

Image, application 1
