# Supplementary figures and images for: Specific inhibition of the NLRP3 inflammasome suppresses immune overactivation and alleviates COVID-19 like pathology in mice
Source: eBioMedicine. 2021 Dec 31;75:103803. doi: 10.1016/j.ebiom.2021.103803 (PMC8719059; doi:10.1016/j.ebiom.2021.103803)

Figure 3A

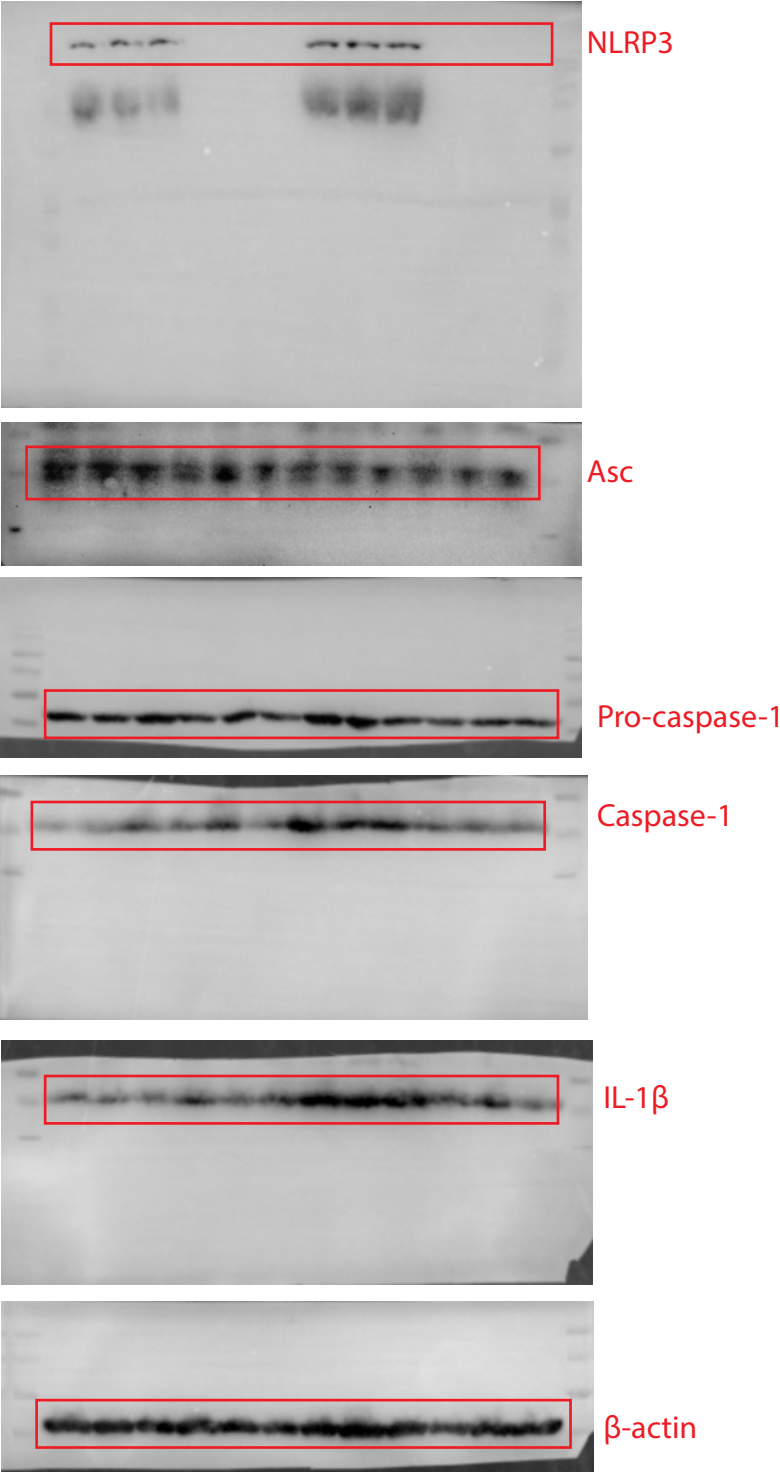

Figure S2A

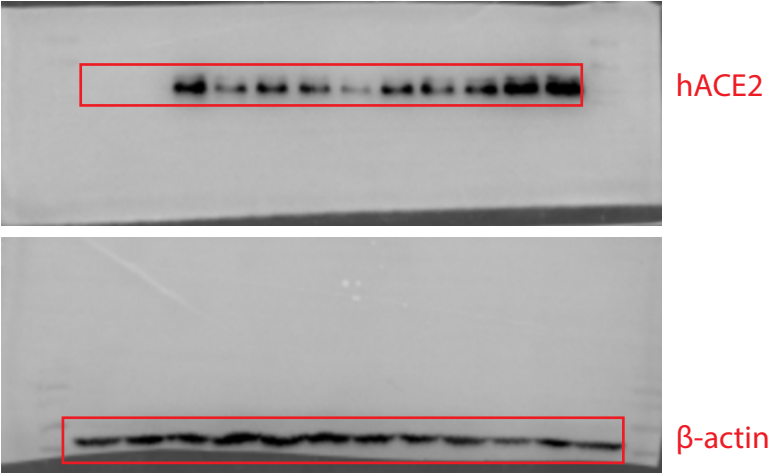

Figure S3A

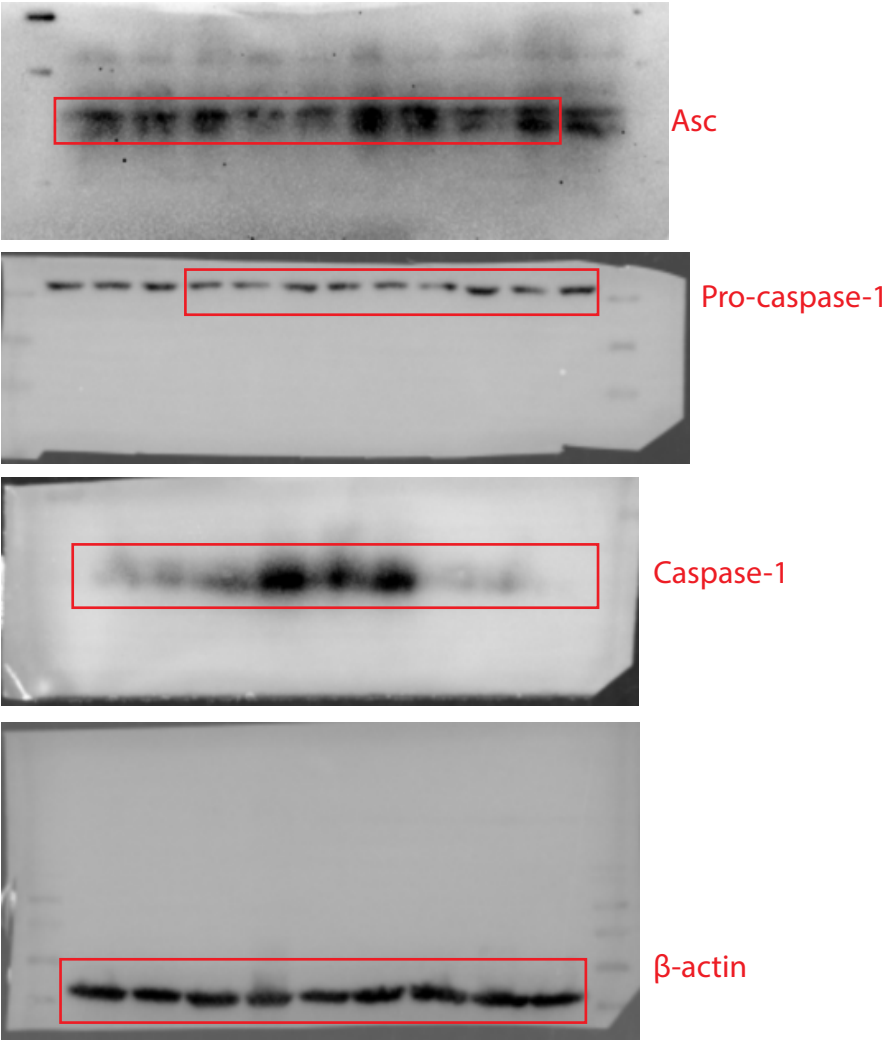

Supplement: Supplementary file 1 [file mmc1.pdf]
